# High-throughput Screening and Sensitized Bacteria Identify an *M. tuberculosis* Dihydrofolate Reductase Inhibitor with Whole Cell Activity

**DOI:** 10.1371/journal.pone.0039961

**Published:** 2012-06-29

**Authors:** Anuradha Kumar, Meng Zhang, Linyun Zhu, Reiling P. Liao, Charles Mutai, Shittu Hafsat, David R. Sherman, Ming-Wei Wang

**Affiliations:** 1 Seattle Biomedical Research Institute, Seattle, Washington, United States of America; 2 The National Center for Drug Screening and State Key Laboratory of Drug Research, Shanghai Institute of Materia Medica, Chinese Academy of Science, Shanghai, China; University of Glasgow, United Kingdom

## Abstract

*Mycobacterium tuberculosis* (*Mtb*), the causative agent of tuberculosis, is a bacterial pathogen that claims roughly 1.4 million lives every year. Current drug regimens are inefficient at clearing infection, requiring at least 6 months of chemotherapy, and resistance to existing agents is rising. There is an urgent need for new drugs that are more effective and faster acting. The folate pathway has been successfully targeted in other pathogens and diseases, but has not yielded a lead drug against tuberculosis. We developed a high-throughput screening assay against *Mtb* dihydrofolate reductase (DHFR), a critical enzyme in the folate pathway, and screened a library consisting of 32,000 synthetic and natural product-derived compounds. One potent inhibitor containing a quinazoline ring was identified. This compound was active against the wild-type laboratory strain H37Rv (MIC_99_ = 207 µM). In addition, an *Mtb* strain with artificially lowered DHFR levels showed increased sensitivity to this compound (MIC_99_ = 70.7 µM), supporting that the inhibition was target-specific. Our results demonstrate the potential to identify *Mtb* DHFR inhibitors with activity against whole cells, and indicate the power of using a recombinant strain of *Mtb* expressing lower levels of DHFR to facilitate the discovery of antimycobacterial agents. With these new tools, we highlight the folate pathway as a potential target for new drugs to combat the tuberculosis epidemic.

## Introduction

Tuberculosis (TB) has a long history in humans, with evidence of TB-related deaths dating back to the 3^rd^ century BC [1]. Arguably one of the oldest and most destructive human diseases, TB continues to be a massive burden on public health around the world. The rise of AIDS (acquired immune deficiency syndrome), and with it a population of people who are more susceptible to opportunistic infections, has escalated the prevalence of TB. Today, roughly 1.4 million people die every year from TB [2], despite the availability of chemotherapies to combat the bacterium that causes this disease (*Mycobacterium tuberculosis, Mtb*). The current standard treatment for tuberculosis is a four-drug combination therapy that lasts a minimum of 6 months [3]. In addition, several factors have led to new strains of *Mtb* that are resistant to many or sometimes all of the first and second line drugs [4]. Depending on the drug susceptibility of the infecting strain, patients must endure prolonged treatment with agents that are more expensive, more toxic, and difficult to administer. The emergence of drug-resistant *Mtb* adds even more urgency to the search for new therapeutics that are better tolerated, more potent and efficacious at eliminating *Mtb* infections in a shorter period of time, and also highlights the importance of focusing on pathways and processes in *Mtb* that are not the targets of existing medications. Recently, there have been increased efforts to develop new and improved therapeutics for tuberculosis, and this has led to a few promising drug candidates [5]. However, it is essential that we continue to develop a pipeline of new classes of compounds that are effective against *Mtb*.

We have focused on the metabolic pathway of folate, which is a critical nutrient in living organisms and is required for the transfer of single-carbon units necessary for many biosynthetic reactions [6], [7]. Tetrahydrofolate (THF) and its derivatives are used as cofactors in the production of thymidylate, purine bases, and some amino acids, such as methionine - essential components of DNA, RNA and protein synthesis. In *Mtb* dihydrofolate reductase (DHFR) is an essential enzyme in this pathway, catalyzing the reduction of dihydrofolate (DHF) into THF. Although DHFR has been extensively studied as an effective drug target in several pathogens and cancer cells, it has not been successfully targeted in *Mtb*: many compounds such as methotrexate and trimethoprim inhibit *Mtb* DHFR *in vitro* but have no effect on the growth of live *Mtb* [8]. However, the promise of this target in other systems has prompted studies focused on developing antifolates that are active against *Mtb*. These efforts were directed towards making analogs of known DHFR inhibitors and exploiting differences in enzyme structures to gain anti-*Mtb* properties [9], [10], [11]. In contrast, we describe here the outcome of a high-throughput screening (HTS) campaign targeting *Mtb* DHFR from which three small molecule inhibitors were identified. Two of these were eliminated in secondary screens, resulting in one compound with specific activity against *Mtb* DHFR. This inhibitor was active against live *Mtb* (MIC_99_ = 207 µM) and appeared to be on-target using a recombinant strain engineered to produce less DHFR. The compound contains a quinazoline ring moiety, a molecular structure similar to the pteridine ring found in many classical folate inhibitors, such as methotrexate. Our results demonstrate the potential for discovery of active molecules to suppress *Mtb* growth by targeting DHFR. They also underscore the promise of screening large and diverse chemical libraries for novel DHFR inhibitors, as well as the need to screen known DHFR inhibitors more thoroughly for compounds with target-specific whole cell activity. Better inhibitors with more potent on-target activity will serve as tools to interrogate the feasibility of targeting DHFR as a therapy for tuberculosis.

## Methods

### Isolation of Mtb Dihydrofolate Reductase (DHFR)

A pET28(a)^+^-derived *E. coli* expression vector containing the *Mtb dfrA* gene with a 6-histidine tag at the amino terminus of the enzyme was generously provided by Dr. J. Sachettini (Texas A&M University) [12], [13]. Recombinant *Mtb* DHFR was expressed and purified essentially as described [12], [13], except that protein expression was induced with 0.2 mM IPTG and harvested cells were resuspended in 20 mM Tris-HCl pH 7.5, 100 mM NaCl, 1 mg/mL lysozyme and EDTA-free protease inhibitors (Sigma, St. Louis, MO) and disrupted by sonication. His-tagged protein was purified by metal affinity chromatography (His-Trap ™ from Amersham, Bucks, UK) followed by size exclusion chromatography (Sepharose 200, Amersham) according to the manufacturers’ instructions. Purified protein was concentrated to between 2–10 mg/mL (20 mM Tris-HCl pH 7.5, 100 mM NaCl and 5% glycerol) in centrifugal filtration units with a 10 kDa molecular weight cutoff (EMD Millipore, Billerica, MA), flash frozen and stored at -80°C.

### Kinetic Assay of DHFR Activity

We used a low-throughput assay for DHFR activity that was modified from previously described assays [14]. The assay contained 0.2 mM NADPH, 10 µM DHF, 8.9 mM ß-mercaptoethanol, 150 mM KCl, 40.7 mM sodium phosphate at pH 7.4 and 125 ng/mL *Mtb* DHFR in a 50 µL volume. Absorbance of NADPH was measured at 340 nm in a 384-well SpectraMax M2 microplate reader (Molecular Devices, Sunnyvale, CA). Activity was calculated as the rate of change in absorbance at 340 nm.

### Diaphorase-coupled Assay of DHFR Activity

The kinetic assay was performed as above, after which 10 µL of a solution containing diaphorase and resazurin was added to give a final concentration of 0.75 U/mL and 0.2 mM, respectively. Fluorescence intensity was measured on SpectraMax M2 microplate reader (Molecular Devices) at an excitation of 560 nm and emission of 590 nm. For the HTS campaign, fluorescent signal was detected by an EnVision plate reader (PerkinElmer, Boston, MA).

### Counter-screening for Activity Against Diaphorase

Diaphorase was diluted to 0.075 U/mL and pre-incubated with 0.2 mM NADPH and varied concentrations of drug in a total volume of 40 µL at room temperature for 5 minutes prior to addition of 5 µL of resazurin to a final concentration of 0.2 mM. After 10 minutes the fluorescence was measured as described above.

### Compound Library

The compound library consisted of 32,000 synthetic and natural compounds and was composed of two sample sources: Novo Nordisk A/S (Bagsværd, Denmark) and SPECS (Delft, Netherlands). The structural diversity covers heterocycles, lactams, sulfonates, sulfonamides, amines, secondary amides and natural product-derived compounds. The compounds were highly purified, and the stock was pre-solubilized in 100% DMSO prior to application in the HTS campaign, performed with an average concentration of 10 µM for each compound.

### Mtb Strains

*Mycobacterium tuberculosis* H37Rv (ATCC 27294) was used as the parent strain in all experiments. In addition, we constructed a DHFR knockdown mutant *Mtb* in which *dfrA* expression is regulated by levels of exogenously added tetracycline. Briefly, we used PCR to produce and amplify a linear fragment encoding (in order): a hygromycin resistance cassette, the tetracycline regulator protein gene *tetR* driven by the constitutive promoter tb21, and a truncated copy of *Mtb dfrA* driven by a tetracycline regulated promoter P_myc1_tetO, which is repressed by TetR in the absence of tetracycline [15]. Following electroporation recombination of the linear DNA fragment into the *Mtb* chromosome at the *dfrA* locus resulted in a single full length copy of *dfrA* under the positive control of a tet-regulated promoter along with a truncated and non-functional copy of *dfrA* (ten amino acids at the carboxy-terminus were deleted). Transformants (H37Rv:*dfrA*-TetON, referred to as DHFR *kd*) were first selected for hygromycin resistance, and successful recombination events were revealed by PCR (primers are listed in Table 1). In the absence of tetracycline the tet promoter is only weakly active, leading to reduced DHFR levels within cells. The strain was maintained in the presence of 50 µg/mL of hygromycin during routine culturing, but was grown in the absence of hygromycin in all other studies detailed below.

**Table 1:** List of primers used in this publication.

Target gene	Primer type	Forward sequence	Reverse sequence	Amplicon (bp)
Rv2763c (dfrA)	outer	cgaggaccaggcgcatttc	tggcggctcagtacgacatttc	129
"	inner	catttccgggagatcaccat	gcagcggccggacttta	85
"	probe	cacatgggattcgct		
Rv2703 (sigA)	outer	aacctgcgcctggtggtttc	ggtgatggcctggcgaatc	174
"	inner	cgcgcctacctcaaacagat	cgtacaggccagcctcgat	91
"	probe	aaggtagcgctgctca		
**PCR validation of H37Rv:** ***dfrA*** **-TetON (DHFR ** ***kd*** **)**				
thyA-hygR	5′ site	gtgccgttcaacatcgccag	cagggattcttgtgtcacag	483
tetO-dfrA	3′ site	tcccggcgttgatctgtgcg	ctcatgagcggtggtagctg	731

### Quantification of Mtb DHFR Transcript Levels

Quantitative PCR (qPCR) was used to determine the levels of *dfrA* RNA in the *Mtb* strains used under various growth conditions. Briefly, RNA was harvested as described previously [16] from wild-type H37Rv and H37Rv:*dfrA*-TetON (DHFR *kd*) cultures grown with varied levels of tetracycline (ATc). Twenty ng of total RNA from each sample was employed to synthesize cDNA using Invitrogen’s Superscript III kit (Carlsbad, CA) following the manufacturer’s instructions. Subsequently, 5 µL of cDNA was used in a two-step qPCR reaction [17]. An initial pre-amplification step was performed with outer forward and reverse primers (all primers are listed in Table 1) and Taq polymerase (Clonetech, Mountain View, CA) with the following program: 95°C for 3 minutes, 15 cycles of 95°C for 20 seconds, 58°C for 1 minute, and 72°C for 1 minute, and a final step at 72°C for 7 minutes. The second qRT-PCR reaction was performed using 0.1 µL of the primary amplification reaction, a set of inner forward and reverse primers, and 6FAM labeled MGB TaqMan probes (Applied Biosystems, Foster City, CA). For each sample, the level of *dfrA* transcript was normalized to that of *sigA* (locus tag: *Rv2703*) generated from the same cDNA preparation.

### Whole-cell Mtb Inhibition Assays

*Mtb* strains were grown in 7H9+/− hygromycin to log phase (OD_600_ = 0.3), diluted to a final OD_600_ of 0.002 and dispensed into a 96-well round-bottom plate (Corning, Acton, MA) in a final volume of 135 µL. To these cells 15 µL of compound diluted in 7H9 and 10% DMSO was added to yield 150 µL in each well at the appropriate final concentration of drug in 1% DMSO (v/v). For each strain, control wells containing no compound were used as a measure of 100% growth, while wells containing a 1∶100 dilution of the starting culture or 1.22 µM rifampicin (rif) were used as a measure of 99% or 100% inhibition, respectively. Plates were incubated at 37°C for 6 days, at which time they were resuspended by pipetting and 20 µL of the total cell mixture was used in a Bac-Titer Glo™ (Promega, Madison, WI) assay of cell viability, as per the manufacturer’s instructions. Luminescence readings were conducted on a Fluo Star Omega plate reader (BMG Lab Tech, Cary, NC). All values were measured from three separate wells and averaged before calculating percent inhibition using the following formula (RLU_no drug_ – RLU_sample_)/(RLU_no drug_ – RLU_rif_). Data from dose-response experiments were analyzed with Graphpad Prism™ (San Diego, CA) and a non-linear least-squares curve was generated to calculate the MIC_99_ for each strain.

## Results

### Development of an HTS-compatible Single-time Point Fluorescent Assay for DHFR

**Figure 1:**
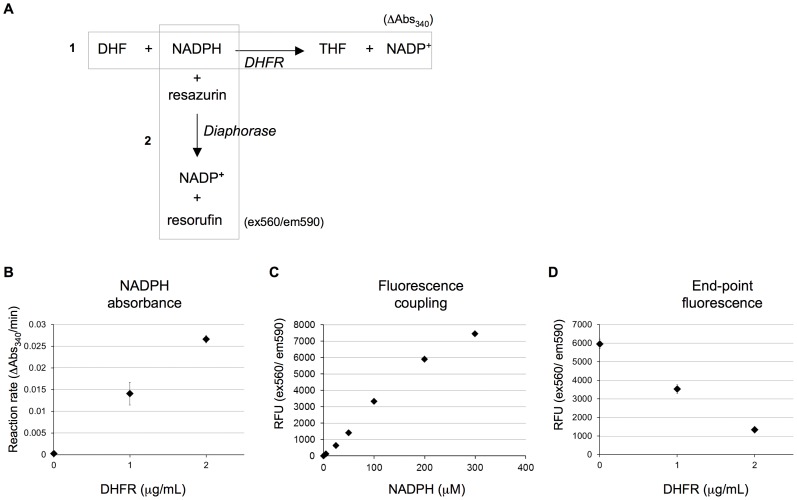
Development of a single-time point assay with a diaphorase-coupled fluorescence readout. A. Reaction schematic. Reaction 1: DHFR, in a NADPH-dependent reaction, converts dihydrofolate (DHF) to tetrahydrofolate (THF), causing a depletion of reduced NADPH. Reaction 2: Diaphorase utilizes NADPH that was unused from the DHFR reaction to generate fluorescence. Enzymes are italicized and bold. B. Kinetic NADPH-depletion assay. The rates of NADPH depletion in reactions performed with 200 µM NADPH, 0.2 mM DHF and varied levels of DHFR were monitored by measuring absorbance at 340 nm. C. Fluorescence coupling. The fluorescence generated after 4 minutes of incubating diaphorase and resazurin with various concentrations of NADPH were measured. D. End-point fluorescence assay. The NADPH-depletion assay run as in (B) was coupled to diaphorase and resazurin after 30 minutes, and the resulting fluorescence measured at excitation and emission wavelengths of 560 and 590 nM, respectively.

The DHFR reaction reduces dihydrofolate (DHF) into tetrahydrofolate (THF) while oxidizing the co-factor NADPH (Figure 1A). NADPH has an absorbance at 340 nm allowing the enzymatic activity of DHFR to be measured by monitoring the rate of depletion of this substrate [14]. We used this kinetic assay to confirm activity of the purified *Mtb* DHFR, to determine the appropriate substrate and enzyme concentrations, and to optimize other assay conditions. Initial titrations indicated that the rate of the reaction is proportional to the amount of DHFR (Figure 1B). However, this assay was not optimal for HTS since it depended on a kinetic readout. Furthermore, small molecules often have absorption spectra overlapping with that of NADPH, complicating measurements and data analyses in that spectral range. Therefore, we adapted the kinetic assay by linking the level of NADPH to a second reaction with a fluorescent readout: the dark blue dye resazurin can be reduced in an NADPH-dependent diaphorase reaction into resorufin, a compound that is bright pink and powerfully fluorescent. First, we titrated the amount of resazurin and diaphorase in the coupling assay to allow a rapid reaction with signal that was proportional to the concentration of NADPH (Figure 1C). We then coupled the kinetic DHFR assay to this end-point fluorescence assay (Figure 1D). The kinetic DHFR reaction was initiated and allowed to proceed, depleting NADPH in solution. After a defined incubation time diaphorase and resazurin were added, causing a reduction of the dye by the remaining NADPH. The kinetic assay was performed with 200 µM NADPH in a 50 µL volume. After the addition of coupling reagents, the final volume of the reaction was 60 µL with a maximal NADPH concentration of 167 µM. As shown in Figure 1C, this was within the linear detection range of the coupled reaction. Hence, the fluorescence in the end-point assay was inversely proportional to the activity of DHFR (Figures 1B and 1D). With no DHFR, the fluorescence signal was high (corresponding to background levels of NADPH depletion). When increasing amounts of DHFR were present the rate of NADPH depletion increased, resulting in a proportional drop in the final fluorescence intensity (Figures 1B and 1D).

### Assay Optimization and Validation for HTS

**Figure 2:**
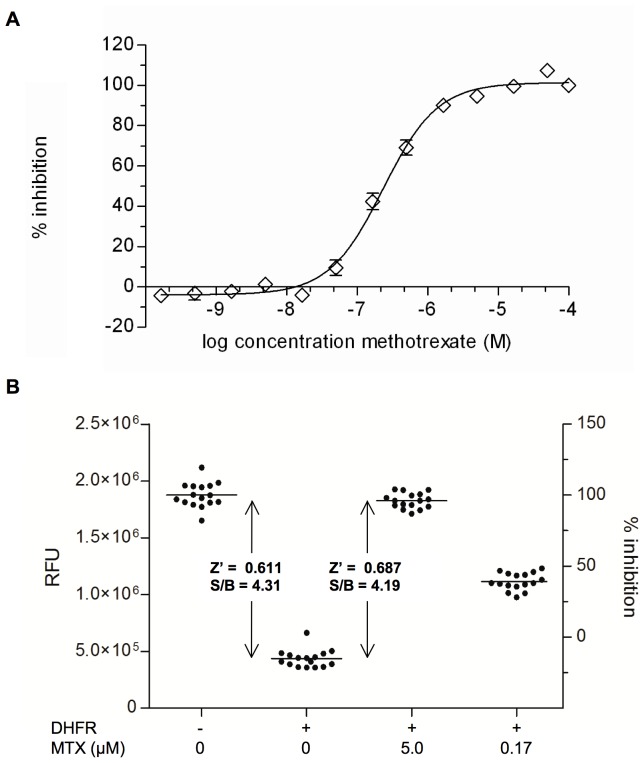
Confirming the robustness of the assay for HTS. A. Standard curve of methotrexate (MTX)**.** The concentration-dependent inhibitory activity of MTX was measured in the coupled assay. B. Statistical Factors. Sixteen replicates of each control reaction were performed in the optimized assay conditions. High-throughput screening (HTS) parameters, including Z’-factor (Z’) and signal to background ratio (S/B) were studied. Raw fluorescence units (right Y-axis) from each of the controls and % inhibition (left Y-axis) are shown. The (-) DHFR controls were used as a measure of complete inhibition, in order to calculate % inhibition.

We tested the robustness of the coupled assay for translation into an HTS format (Figure 2) using methotrexate (MTX, a potent inhibitor of purified *Mtb* DHFR) as a control compound while developing and validating the assay. We consistently found a good dose response to MTX with an IC_50_ of 237 nM (Figure 2A). This value exceeds the previously published value of 8.3 nM [14] because relatively high substrate concentrations were used. Based on these results we found that 5 µM MTX consistently resulted in full inhibition of *Mtb* DHFR in this assay (Figures 2A and 2B).

Reactions performed with carrier DMSO alone were a measure of full uninhibited *Mtb* DHFR activity, and served as a negative control equivalent to 0% inhibition. Reactions performed in the absence of *Mtb* DHFR were equivalent to complete inhibition and served as positive controls (Figure 2B). Studies were repeated on three separate days, with all mixes being freshly made, to evaluate the variation between days. Plates were run with 2 sets of controls, each with 16 replicates per plate. When performed in 384-well plates a Z’ factor of 0.611 and S/B value of 4.31 were recorded with CV values of 5.8% and 17.9% for the positive and negative controls, respectively. However, to simplify the liquid handling steps during the HTS campaign we used reactions conducted with 5 µM MTX as a positive control, since we had previously established this as a proxy for reactions lacking enzyme. We confirmed that the dynamic range and robustness of the assay using these controls were very similar, yielding a Z’ factor of 0.687 and S/B value of 4.19 (Figure 2B). These statistical parameters indicated that the assay met HTS requirements [18].

We also studied the stability of the reagents. At each time point three sets of control reactions were performed: *Mtb* DHFR with no inhibitor (DMSO alone), *Mtb* DHFR with 5 µM MTX, and the negative control reaction in the absence of *Mtb* DHFR. Over 150 minutes we saw only very low levels of NADPH auto-oxidation (2.42% loss as measured by a decrease in light absorbance at 340 nm; Figure S1). Similarly, we found that the magnitude of the fluorescent signals for both the positive and negative control reactions dropped very slightly, corresponding to the NADPH auto-oxidation. However, the difference between the positive and negative signals remained constant, indicating that the total NADPH was still in sufficient excess and that the rate of the *Mtb* DHFR reaction was not affected over the 150 minutes (Figure S1). During the HTS campaign fresh solutions were prepared every 3 hours to ensure that the total NADPH present in solution was sufficient.

### HTS Campaign and Confirmation Studies

**Figure 3:**
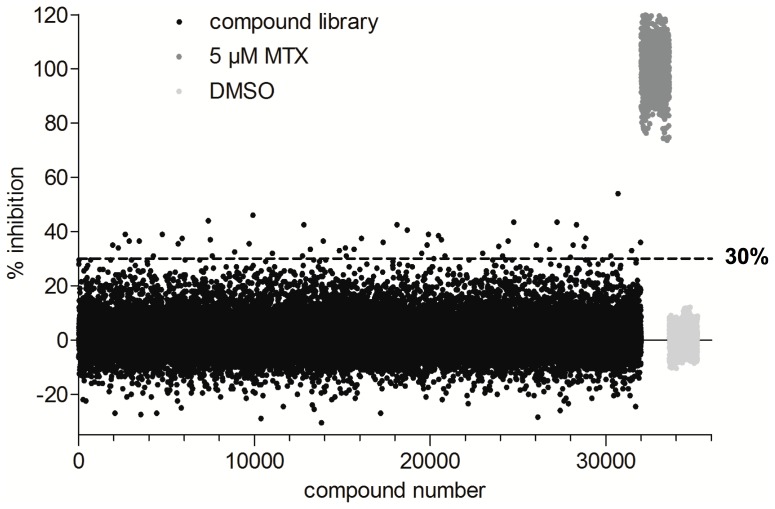
Primary screening. A collection of 32,000 compounds were tested against the optimized DHFR assay at a final concentration of 10 µM. Sixteen positive (5 µM MTX shown in pale grey symbols) and negative (DMSO alone shown in dark grey symbols) control reactions were plated on each of the 100 screening plates in order to calculate % inhibition. Controls from the entire screening are shown clustered together. An initial threshold for hits was set at 30% inhibition (dashed line).

**Figure 4:**
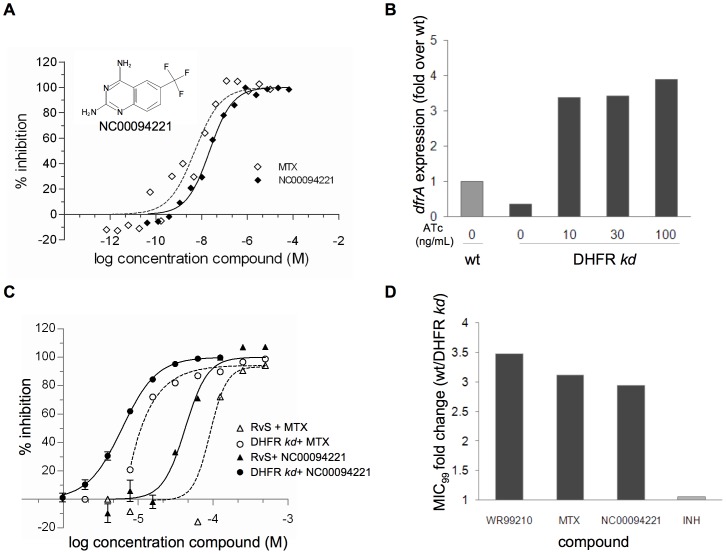
Characterization of NC00094221. A. Dose-response curves in low-throughput *Mtb* DHFR enzyme assay. NC00094221 (chemical structure shown inset into the graph) and MTX were tested at varied concentrations in the kinetic assay performed with 10 µM DHF and 125 ng/mL recombinant DHFR. Data were fitted to a non-linear least-squares curve and IC_50_ values were calculated using GraphPad Prism™.**B**. Quanitification of DHFR transcript levels in a recombinant *Mtb* strain. The levels of DHFR transcript were assayed by qRT-PCR in wild-type *Mtb* strain H37Rv (wt- pale grey bar) and the engineered *Mtb* strain (H37Rv:*dfrA*-TetON, referred to as DHFR *kd*- dark grey bars) in the presence of varied levels of tetracycline (ATc). DHFR transcript levels from each sample were first normalized to SigA transcript levels and then shown as the fold change when compared to wild-type (wt) *Mtb*. C. Live *Mtb* growth inhibition assay. Varied concentrations of methotrexate (MTX, white symbols) or NC00094221 (black symbols) were incubated with wild-type *Mtb* (triangles) or the DHFR *kd* (circles) and grown for 6 days prior to assessment of cell growth using the commercially available Bactiter-Glo assay kit. For each strain, controls including wells with no drug (DMSO carrier was added instead), 1.22 µM rifampicin (10× MIC_99_) and a 1/100 dilution of the starting culture were used to calculate 0%, 100% and 99% inhibition, respectively. Data were fitted to a non-linear least-squares curve and MIC_99_ calculations were performed using GraphPad Prism™. D. Target-specific drug sensitivity in DHFR *kd*. The sensitivity of the wild-type *Mtb* and DHFR *kd* was tested against various inhibitors and displayed as the ratio of a compound’s MIC_99_ when measured in wild-type *Mtb* over the MIC_99_ measured in DHFR *kd* in the absence of ATc.

We then implemented an HTS campaign against a 32,000- compound library. The screen was conducted over four days, with 25 plates screened per day. In each 384-well plate, one column on each edge was reserved for positive (5 µM MTX) or negative controls (DMSO). They showed good reproducibility across plates and between different screening days (Figure 3). The CV between all the control wells from the 100 plates was 6.4% and 12.2% for the positive and negative controls, respectively. Inhibition of *Mtb* DHFR was calculated from the controls within each individual plate. In the initial screen, 52 ‘hits’ (0.16%) displayed greater than 30% inhibition (Figure 3). Selected hits were re-screened at a concentration of 10 µM and three compounds consistently inhibited *Mtb* DHFR activity in this assay. Confirmed hits (0.01%) were further tested for concentration-dependent response characteristics (Figure S2.A). All three compounds exhibited inhibition in a dose-dependent manner and were further examined in the counter-screen for activity against the coupling enzyme, diaphorase (Figure S2.B). Two of the compounds inhibited diaphorase, indicating that their activity was not specific for DHFR. NC00094221 (inset in Figure 4A) had no inhibitory activity against the counter-screen. All three compounds were next evaluated in the low-throughput assay against *Mtb* DHFR. As expected, the two inhibitors active in the counter-screen were not active in this assay (data not shown), while NC00094221 displayed specific activity against *Mtb* DHFR, with an estimated IC_50_ of 22.4 nM. In comparison, MTX showed an IC_50_ of 5.1 nM (Figure 4A).

### In-vitro Activity Against Live Mtb

After establishing the activity of NC00094221 against purified recombinant enzyme, we sought to determine if it could inhibit the growth of whole-cell *Mtb*. We also wanted to address two factors complicating our ability to interpret results of tests performed on wild-type *Mtb*: the possibility that compounds were unable to pass through the cell wall as well as the potential off-target effect. As described earlier, many inhibitors with potent activity against purified recombinant *Mtb* DHFR such as MTX fail to show activity in whole cells. If compound penetration was poor, then medicinal chemistry might be employed to increase intracellular concentrations. However, the *in vivo* target would still be unclear. We reasoned that a strain with artificially lowered enzyme could address both issues of cell permeability and target specificity [19], [20]. Lowering the levels of DHFR would sensitize bacteria to folate pathway inhibitors and conversely we would expect wild-type *Mtb* to be more resistant to inhibition by the same concentration of an active compound.

The use of tetracycline derivatives to regulate gene expression has been well studied in bacteria and has been successfully adapted for mycobacterial gene regulation [15]. We utilized a single-cross over homologous recombination strategy to replace the wild-type promoter of *Mtb dfrA* with a tetracycline regulated promoter, which yielded the partial merodiploid strain H37Rv:*dfrA*-TetON (DHFR *kd*) containing a full length *Mtb* DHFR under the positive control of anhydro-tetracycline (ATc). DHFR *kd* exhibited only a very slight growth defect *in vitro* that was rescued when cultured in the presence of ATc (data not shown). We measured *dfrA* transcript levels in this strain under varied growth conditions (Figure 4B). Without ATc, levels of *dfrA* transcript were 2.8-fold lower than that of wild-type *Mtb*, but in the presence of 100 ng/mL ATc transcription of *dfrA* increased by 3.9-fold (Figure 4B). DHFR *kd* should be hypersusceptible only to inhibitors that target the folate pathway. We determined that there was no significant change in the MIC_99_ of isoniazid (INH) between the two strains (Figure 4D), which targets cell wall synthesis [21]. However, using a previously published inhibitor of *Mtb* DHFR, WR99210 [10], we showed that in the absence of ATc DHFR *kd* is approximately three times more susceptible to growth inhibition compared with H37Rv (Figure 4D). Further studies with DHFR *kd* confirmed that MTX, while barely active against wild-type *Mtb*, had more convincing concentration-dependent whole-cell inhibitory activity in the DHFR-sensitized strain (Figure 4C). The MIC_99_ of MTX against wild-type *Mtb* was over 3-fold higher than against the sensitized strain (Figure 4D), suggesting that it targets DHFR or the folate pathway in live cells. We then tested the active compound identified from our primary screen for activity on DHFR *kd*. We found that the potency of NC00094221 increased by about 3-fold in the knockdown strain (MIC_99_ of 207 µM in H37Rv *vs*. MIC_99_ of 70.7 µM in DHFR *kd*; Figures 4C and 4D), demonstrating that DHFR or a related enzyme in the folate pathway is the relevant intracellular target of this compound.

## Discussion

Reduced folate is a critical nutrient in living organisms, and disruption of the folate pathway is widely exploited for chemotherapy [6], [22]. Dihydrofolate reductase is one of the most commonly studied enzymes in the folate pathway and is a well-established drug target. MTX, developed in the 1950’s as an inhibitor of DHFR, has historically been prescribed to treat cancers and skin diseases [6]. Pyrimethamine and trimethoprim, also inhibitors of DHFR, are more selective for protozoal and bacterial DHFR, respectively, compared to the human enzyme, and have routinely been applied to treat malaria and as general antimicrobials [6], [23]. The efficacy of these therapeutics and their analogs is enhanced in combination with agents that target dihydropteroate synthetase (DHPS), another folate pathway enzyme that is unique to prokaryotes [8]. While these combinations have not yet yielded a treatment for tuberculosis, they are potent against *Mycobacterium avium* and *Mycobacterium leprae*, two close relatives of *Mtb* [24]. Clearly, an opportunity exists to develop an active inhibitor of *Mtb* DHFR with clinical value against tuberculosis.

Several groups have adapted previously discovered DHFR inhibitors in an effort to improve selectivity for *Mtb* DHFR and to increase potency against live *Mtb* [10], [11]. A parallel approach would be to find active molecules with novel chemical structures. This would provide a broader base of scaffolds to be used as starting points for drug discovery. To this end, we developed an HTS-compatible assay to search for novel inhibitors of *Mtb* DHFR. It was employed to screen a compound library of 32,000 synthetic and natural molecules. Of the hits identified and confirmed, NC00094221 demonstrated a dose-dependent inhibition on the purified recombinant *Mtb* DHFR (IC_50_ = 22.4 nM), as well as DHFR-specific growth suppression of live *Mtb*, displaying an MIC_99_ of 207 µM.

NC00094221 belongs to the diaminoquinazoline family of compounds, and its characterization as an *Mtb* DHFR inhibitor from this library is consistent with previous research that identified many classical folate inhibitors containing quinazoline rings [25]. The latter are related to pteridine rings seen in both the DHFR substrate, dihydrofolate, and the drug, MTX. When compared to MTX, NC00094221 showed weaker inhibition of the purified *Mtb* enzyme (NC00094221 IC_50_ = 22.4 nM *vs*. MTX IC_50_ = 5.1 nM, Figure 4A). However, NC00094221 was more potent against whole-cell *Mtb* (Figure 4C). Our results confirmed that this activity was directed at the folate pathway in live *Mtb* since the *dfrA* knockdown strain was more susceptible (Figures 4C and 4D). In addition, a previous study identified this chemical structure as an active inhibitor of the DHFR enzymes isolated from *P. carinii* (IC_50_ = 2.7 µM) and *T. gondii* [25], but its potency against *Mtb* DHFR or whole-cell *Mtb* was not reported. These data suggest that a more thorough search of other previously identified antifolates may reveal potent antiymcobacterial agents with DHFR-specific activity.

While previously identified inhibitors of the *Mtb* DHFR enzyme such as WR99210 and its analogs have displayed on-target activity against live *Mtb* [10], they proved unsuitable for testing in animals because of high toxicity to host cells. Thus, the challenge has been to find a compound with high specificity for *Mtb* DHFR that has little or no activity against the human enzyme. Our work indicates that a more thorough analysis of known antifolates might be a valuable method in the search for DHFR-specific antimycobacterial agents. It also underscores the value of target-based whole-cell assays using knockdown strains for identifying compounds with potent on-target activity against live pathogens such as *Mtb*. Many potent enzyme inhibitors fail in whole-cell assays, in part due to their poor permeability through the notoriously waxy outer coat of *Mtb*. Evaluating compounds against wild-type *Mtb* alone sets a high bar for compounds that may have limited cell entry. It also removes from consideration compounds that have specific activity against the enzyme but possess weak or no activity against the whole cell. Conversely, determining the ability of compounds to modulate the growth of *Mtb* made hyper-susceptible to DHFR inhibition permits us to identify weaker molecules, thereby gaining valuable information concerning structure-activity relationship to assist the design of effective drug leads against *Mtb*. With the growing demand for better anti-tuberculosis agents, it is incumbent on us to pursue diverse avenues of drug discovery. Focusing on an area such as the folate pathway, with its rich history of successful drugs, is therefore a promising way forward.

## Supporting Information

Figure S1 **Stability of assay reagents.** A single batch of reagents was used in the HTS assay at 0, 50, 100 and 150 minutes (8 wells for each control) after having been freshly prepared and left at room temperature. The mean at each time point as well as the average of the measurements over 150 minutes. (32 replicates total) are plotted.
(TIF)

Figure S2 A. Dose response curve in primary HTS assay. The dose-response characteristics of the three hits identified from the library were studied in the diaphorase-coupled enzyme assay. Methotrexate (MTX) was used as a positive control. B. Counter-screening. The activity of the three hits were tested against the coupling enzyme (diaphorase) used in the high-throughput screen (HTS). Only NC00094221 did not inhibit the counter-screen.
(TIF)
