# Supplementary figures and images for: High-throughput Screening and Sensitized Bacteria Identify an M. tuberculosis Dihydrofolate Reductase Inhibitor with Whole Cell Activity
Source: PLoS One. 2012 Jun 29;7(6):e39961. doi: 10.1371/journal.pone.0039961 (PMC3386958; doi:10.1371/journal.pone.0039961)

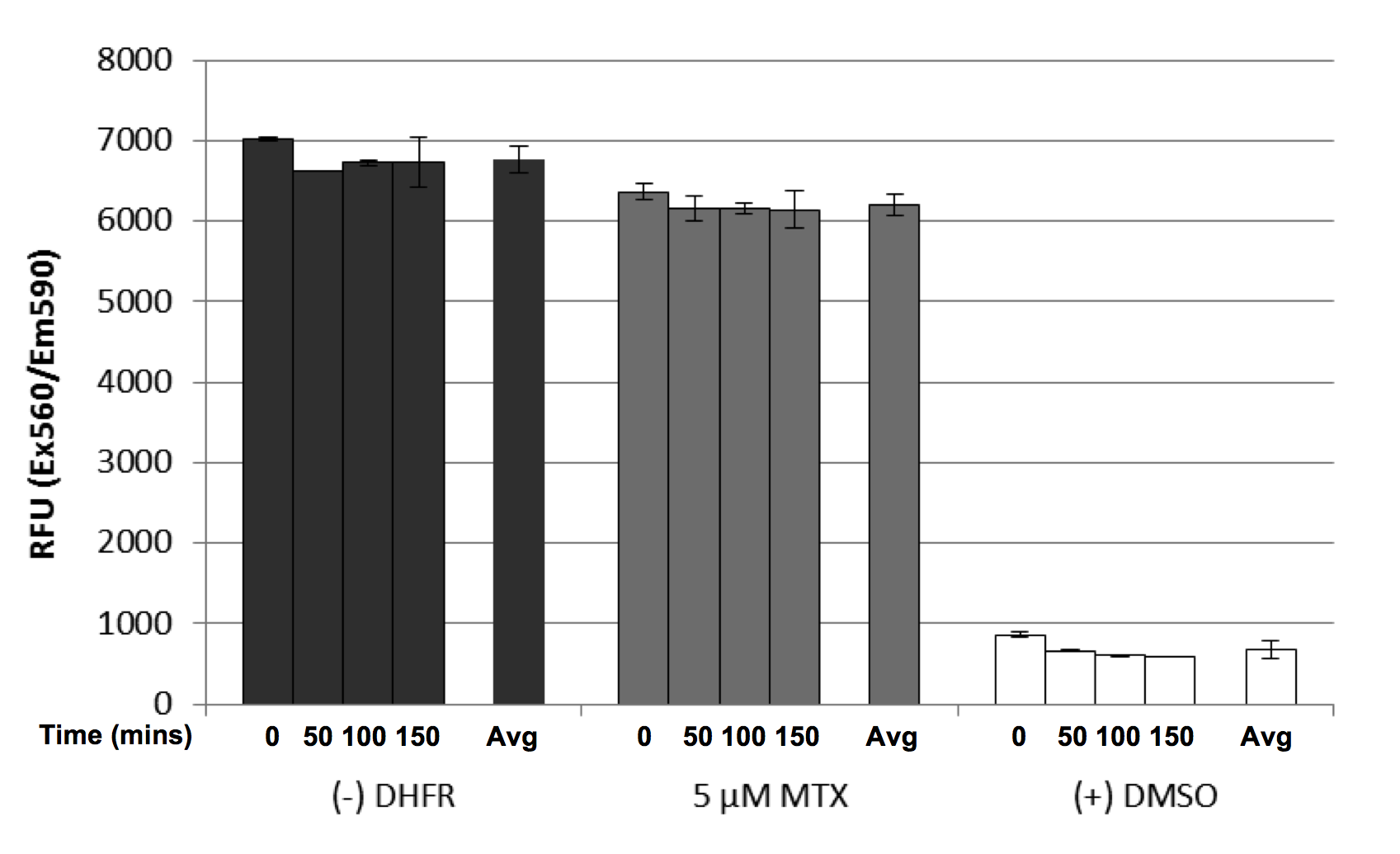

Supplement: Figure S1 — Stability of assay reagents. A single batch of reagents was used in the HTS assay at 0, 50, 100 and 150 minutes (8 wells for each control) after having been freshly prepared and left at room temperature. The mean at each time point as well as the average of the measurements over 150 minutes. (32 replicates total) are plotted. (TIF) [file pone.0039961.s001.tif]

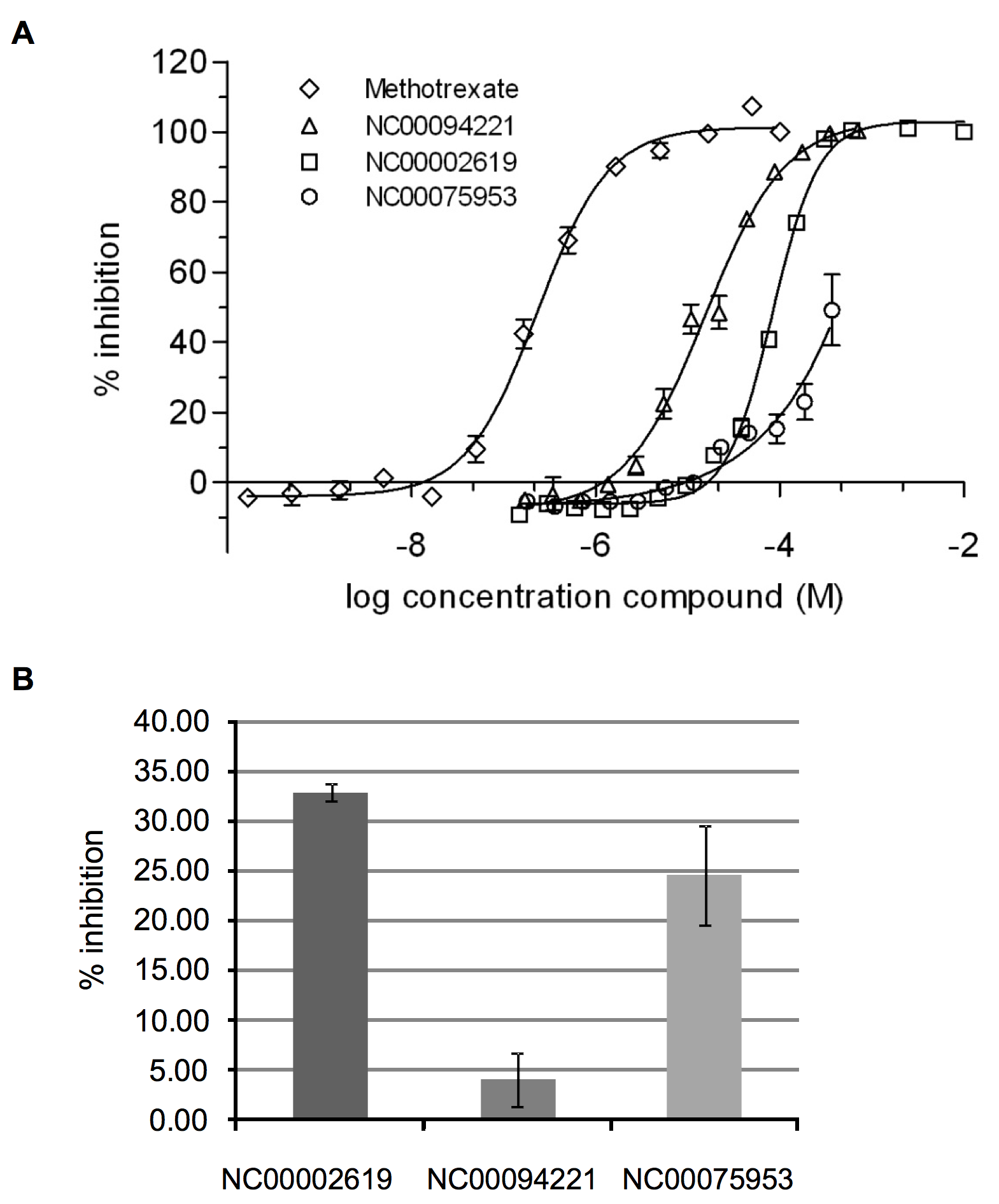

Supplement: Figure S2 — A. Dose response curve in primary HTS assay. The dose-response characteristics of the three hits identified from the library were studied in the diaphorase-coupled enzyme assay. Methotrexate (MTX) was used as a positive control. B. Counter-screening. The activity of the three hits were tested against the coupling enzyme (diaphorase) used in the high-throughput screen (HTS). Only NC00094221 did not inhibit the counter-screen. (TIF) [file pone.0039961.s002.tif]
